# Sex-Specific Differences in the Relationship Between Prematurity and Ocular Geometry

**DOI:** 10.1167/iovs.65.6.23

**Published:** 2024-06-14

**Authors:** Achim Fieß, Alica Hartmann, Eva Mildenberger, Michael S. Urschitz, Panagiotis Laspas, Anna Schultheis, Bernhard Stoffelns, Norbert Pfeiffer, Sandra Gißler, Alexander K. Schuster

**Affiliations:** 1 Department of Ophthalmology, University Medical Center of the Johannes Gutenberg University Mainz, Mainz, Germany; 2 Division of Neonatology, Department of Pediatrics, University Medical Center of the Johannes Gutenberg University Mainz, Mainz, Germany; 3 Division of Pediatric Epidemiology, Institute for Medical Biostatistics, Epidemiology and Informatics, University Medical Center of the Johannes Gutenberg University Mainz, Mainz, Germany

**Keywords:** prematurity, epidemiology, birth weight, anatomy, sex differences

## Abstract

**Purpose:**

To explore differences in the relationship between gestational age (GA) and birth weight (BW) percentile and ocular geometry between males and females.

**Methods:**

The Gutenberg Prematurity Eye Study involved a prospective ophthalmic examination of adults, aged 18 to 52 years, who were born preterm or at term, in Germany. The associations between GA and BW percentile on the main outcome measures were evaluated by uni- and multivariable linear regression analyses. The main outcome measures were central corneal thickness, corneal radius, anterior chamber depth, lens thickness, posterior segment length, and central foveal thickness. Potential sex-specific differences and an effect modification by sex were analyzed.

**Results:**

This study involved 438 participants (245 females, 193 males) with an average age of 28.6 ± 8.7 years. In female participants, central foveal thickness was negatively associated with a higher GA (*B* = −2.99; *P* < 0.001). Similarly, male participants also demonstrated a negative association between central foveal thickness and GA (*B* = −4.27; *P* < 0.001). The multivariable model with effect modification revealed that the central foveal thickness was thicker with lower GA. There was an association between the effect modification of GA with sex and central foveal thickness, demonstrating a more pronounced effect of GA on central foveal thickness in male participants (*B* = 1.29; *P* = 0.04).

**Conclusions:**

This study identified a sex-specific correlation between lower GA and thicker central foveal thickness, suggesting differences in the developmental trajectory of this biometric parameter concerning GA. A thicker central foveal thickness might affect the visual acuity of individuals born preterm in adulthood, with a more pronounced impact in males and a potential predisposition to age-related diseases later in life. Sex did not influence the association of GA or BW percentile to other ocular geometric parameters.

Numerous studies have demonstrated disparities in ocular biometry between infants born preterm with and without retinopathy of prematurity (ROP) compared to individuals born full-term contributing to the increased risk for reduced visual acuity and increased myopic refractive errors of individuals born preterm in infancy and childhood.^1^^–^^3^ Furthermore, there are biometric differences between men and women in adults.^4^^,^^5^ However, the expression of these biometric differences between the sexes in children born preterm remains unclear, raising questions about the existence of sex-specific differences regarding ocular biometry. There is evidence that preterm birth affects women and men differently in various aspects, with sex-specific differences becoming evident during fetal development. Female fetuses rely more on intrinsic placental growth, whereas male fetuses depend more on nutrient transfer through the placenta. Consequently, in times of maternal stress and deprivation, male fetuses are at a higher risk of experiencing growth retardation, including reduced growth.^6^^–^^8^ Moreover, male preterm infants have an elevated risk of mortality, morbidity, and brain injury,^9^^–^^11^ and certain studies have indicated a higher incidence of severe ROP in male individuals.^12^^,^^13^ The long-term effects of prematurity also appear to differ between female and male individuals, with several studies indicating that females born preterm have better cognitive outcomes compared to males.^14^^–^^16^

Sex differences in ocular biometry in children as well as in adults have been investigated. In a study examining 155 eyes, female adults had a thinner central corneal thickness, but the axial length, anterior chamber depth, lens thickness, and white-to-white distance were not significantly different between sexes.^17^ Conversely, other studies have demonstrated that males tend to have larger morphometric eye parameters. In one study, sex-specific biometric differences in children were examined by analyzing 64 eyes of 64 adolescents from 32 sets of twins.^18^ The authors discovered sex-specific variations in optical biometry measurements. It was observed that girls were more likely to show shorter axial lengths and white-to-white distances.^18^ Other studies in adults have shown comparable results related to generally larger morphometric parameters in men.^4^^,^^5^

In a prior investigation conducted as part of the Gutenberg Prematurity Eye Study, it was demonstrated that low gestational age (GA) and a low birth weight (BW) percentile were linked to a steeper corneal curvature and a reduced corneal diameter. Meanwhile, ROP was solely associated with reduced corneal diameter, with ROP-treated eyes exhibiting a shallower anterior chamber and a thicker lens. Additionally, lower GA and the presence of pre-eclampsia were linked to a shorter axial length.^19^ Other studies have yielded similar findings.^20^^–^^22^

This study aims to build upon the previous findings by exploring sex-specific differences in ocular biometry, with a primary focus on individuals born preterm. This study represents the first of its kind, to our knowledge, to investigate disparities in adults born preterm, and it is anticipated that it will uncover sex-specific variations that could impact vision. Understanding these potential sex-specific differences in ocular biometry could provide valuable insights for clinical practice.

## Materials and Methods

### Study Population

The Gutenberg Prematurity Eye Study (GPES) is a single-center cohort study conducted at the University Medical Center of the Johannes Gutenberg University Mainz (UMCM) in Germany. The study included individuals born preterm or full term between 1969 and 2002 who were between the ages of 18 to 52 years at the time of enrollment. This study has a retrospective cohort design with prospective follow-up data collection.

For the GPES, preterm newborns with a GA at birth of ≤32 weeks and every second randomly chosen preterm newborn with a GA of 33 to 36 weeks were invited to participate. Additionally, for each month from 1969 to 2002, six individuals (three males and three females) born full term with a BW between the 10th and 90th percentiles were invited as controls, as reported earlier.^23^^,^^24^

The study examinations were conducted from June 2019 to November 2021. Each participant underwent a comprehensive ophthalmological examination, including ocular biometry, and a medical history interview. Furthermore, the participants’ medical records documenting perinatal and postnatal histories were reviewed.

Written informed consent was obtained from all participants before they entered into the study, complying with Good Clinical Practice, Good Epidemiological Practice, and the tenets of the Declaration of Helsinki. The study protocol and documents were approved by the local ethics committee of the Medical Chamber of Rhineland-Palatinate, Germany (reference no. 2019-14161; original vote: 29.05.2019, latest update: 02.04.2020).

### Assessment of Prenatal, Perinatal, and Postnatal Medical History

The medical records of the participants archived at the UMCM were thoroughly reviewed to gather essential information related to their prenatal, perinatal, and postnatal medical history. Data included parameters such as GA (in weeks); BW (in kilograms); the presence and stage of ROP and ROP treatment details; occurrences of placental insufficiency; cases of pre-eclampsia; breastfeeding practices; gestational diabetes; maternal hemolysis, elevated liver enzymes, low platelet count (HELLP) syndrome; and maternal smoking. Additionally, BW percentiles were calculated following the method developed by Voigt et al.^25^

### Categorization

The participants were categorized into the following groups for descriptive analysis:

• Group 1. Full-term participants (born with a GA of ≥37 weeks)
• Group 2. Preterm participants with a GA at birth between 33 and 36 weeks (moderate-to-late preterm) without ROP
• Group 3. Preterm participants with a GA at birth between 29 and 32 weeks without ROP
• Group 4. Preterm participants with a GA at birth of ≤28 weeks without ROP
• Group 5. Preterm participants with a GA at birth of ≤32 weeks with postnatal ROP but no ROP treatment
• Group 6. Preterm participants with a GA at birth of ≤32 weeks with postnatal ROP and ROP treatment

In cases where only one eye of a participant had ROP, the analysis excluded the fellow eye without ROP.

### Ophthalmological Examination

Ocular biometry was conducted with the Lenstar 900 instrument from Haag-Streit (Köniz, Switzerland).^23^ During each examination, three individual measurements were taken, and the average value was calculated. Several Lenstar parameters were recorded, including corneal radius, anterior chamber depth, lens thickness, and axial length. Each parameter underwent outlier checks to ensure data quality as reported earlier.^19^ Furthermore, nonmydriatic fundus photography and imaging of the macula using spectral-domain optical coherence tomography (SD-OCT) were conducted with the SPECTRALIS OCT system (Heidelberg Engineering, Heidelberg, Germany).^19^^,^^26^ The macula was captured using SD-OCT in a 15° × 15° block scan format in enhanced depth imaging mode and assuming a corneal curvature of 7.7 mm. Heidelberg Eye Explorer software (HEYEX and SPX; Heidelberg Engineering) was employed, utilizing a research software tool for the automatic segmentation of macular retinal thickness and that of each individual retinal layer. The calculation of retinal layer thickness within the fovea and perifoveal area was executed using an Early Treatment Diabetic Retinopathy Study (ETDRS) grid featuring circles at 1-mm, 2-mm, and 3-mm distances from the foveal center, which was presumed to be the point of deepest foveal depression. Central foveal thickness was determined as the minimal retinal thickness at the fovea. Each scan underwent review by a board-certified ophthalmologist to identify any issues with decentration or layer segmentation errors, with any affected eyes being excluded from the study. Consequently, this study incorporated only those images that were of high quality and perfectly centered, possessed a signal strength of over 15 dB, and showcased precise automated delineation.^27^ Moreover, foveal scans from both eyes were assessed by two independent graders (A.K.S., S.G.) for signs of foveal hypoplasia, defined per criteria outlined in a prior publication.^26^

### Covariates

Covariates may affect the main outcome measures such as GA (weeks) and BW percentile, so participants with a history of corneal or cataract surgery were excluded, as such medical interventions could potentially impact ocular anatomy (*n* = 7). Also, ocular geometry measurements were not possible in a few participants, particularly in those with low visual acuity (*n* = 5).

### Statistical Analysis

The main outcome measures were central corneal thickness, corneal radius, anterior chamber depth, lens thickness, posterior segment length, and central foveal thickness. Descriptive statistics were utilized to analyze these outcome measures stratified by clinical group. For dichotomous parameters, absolute and relative frequencies were calculated, whereas approximately normally distributed variables were assessed using mean and standard deviation (SD) and the median and interquartile range were determined for non-normally distributed variables. Both right and left eyes were included in the analysis. Linear regression models with generalized estimating equations were employed to evaluate associations accounting for correlations between corresponding eyes.

The initial step involved univariable analyses for the main outcome measures considering independent parameters such as GA and BW percentile. The regression analyses were conducted independently for female and male participants to explore potential sex-specific differences. Both GA and BW percentile were incorporated into the multivariable regression analysis concerning various biometric parameters. An additional model was introduced to investigate the potential influence of sex, employing effect modification terms in the regression model. Moreover, a sensitivity analysis was performed by incorporating age into the multivariable model for central foveal thickness. Further, a separate multivariable analysis was conducted adjusting for the presence of ROP (yes) and ROP treatment (yes) as additional factors. In a separate model, foveal hypoplasia was included as a further adjustment. Because this study is exploratory, no adjustments for multiple testing were applied. The calculations were performed using commercial software (SPSS Statistics 20.0; IBM, Chicago, IL, USA).

## Results

A total of 861 eyes of 438 individuals, including 245 females and 193 males, were included in this study with an average age of 28.6 ± 8.7 years. Among the female participants, 81 individuals had a GA of 37 weeks or more (group 1), 80 individuals had a GA between 33 and 36 weeks without ROP (group 2), 47 individuals had a GA between 29 and 32 weeks without ROP (group 3), 10 individuals had a GA of 28 weeks or less without ROP (group 4), 23 individuals had a GA between 24 and 32 weeks with ROP but without treatment (group 5), and four individuals had a GA between 24 and 32 weeks with postnatal treatment for ROP (group 6) (Table 1).

**Table 1.** Characteristics of the GPES Sample (*n* = 438) Stratified by Study Group and Grouped by Female and Male Participants

	Group 1 (GA ≥ 37)	Group 2 (GA 33–36) No ROP	Group 3 (GA 29–32) No ROP	Group 4 (GA ≤ 28) No ROP	Group 5 (GA ≤ 32) ROP Without Treatment	Group 6 (GA ≤ 32) ROP With Treatment
Female participants						
Participants/eyes, *n*	81/162	80/159	47/94	10/18	23/44	4/7
Age (y), mean ± SD	29.40 ± 8.9	29.28 ± 9.7	28.66 ± 8.7	28.50 ± 10.5	23.65 ± 6.5	24.25 ± 5.2
BW (g), mean ± SD	3320 ± 375	2090 ± 487	1520 ± 282	1017 ± 182	933 ± 344	844 ± 228
BW < 1500 g, *n* (%)	0 (0)	7 (8.8)	19 (40.4)	10 (100)	21 (91.3)	4 (100)
BW < 1000 g, *n* (%)	0 (0)	0 (0)	3 (6.4)	4 (40.0)	14 (60.9)	3 (75.0)
GA (wk), mean ± SD	39.44 ± 1.3	34.43 ± 1.0	30.51 ± 1.2	27.00 ± 1.3	27.57 ± 2.0	26.25 ± 2.1
ROP, stages 1/2/3, *n*	0/0/0	0/0/0	0/0/0	0/0/0	17/25/2	0/2/5
Preeclampsia, *n* (%)	9 (11.1)	13 (16.3)	7 /14.9)	1 (10.0)	4 (17.4)	0 (0)
Placental insufficiency, *n* (%)	2 (2.5)	13 (16.3)	2 (4.3)	0 (0)	1 (4.3)	0 (0)
Maternal smoking, *n* (%)	5 (6.2)	6 (7.5)	4 (8.5)	1 (10.0)	3 (13.0)	0 (0)
HELLP syndrome, *n* (%)	0 (0)	2 (2.5)	0 (0)	0 (0)	2 (8.7)	0 (0)
Gestational diabetes, *n* (%)	1 (1.2)	4 (5.0)	0 (0)	0 (0)	0 (0)	0 (0)
Breastfeeding, *n* (%)	43 (53.1)	45 (56.3)	23 (48.9)	3 (30.0)	10 (43.5)	3 (75.0)
Male participants
Participants/eyes, *n*	59/118	54/108	41/81	10/18	20/36	9/16
Age (y), mean ± SD	30.49 ± 9.6	29.63 ± 8.6	27.80 ± 7.5	20.90 ± 3.1	25.90 ± 4.1	28.44 ± 5.3
BW (g), mean ± SD	3558 ± 376	2023 ± 440	1582 ± 377	835 ± 156	1166 ± 411	803 ± 288
BW < 1500 g, *n* (%)	0 (0)	6 (11.1)	18 (43.9)	10 (100)	16 (80)	9 (100)
BW < 1000 g, *n* (%)	0 (0)	0 (0)	2 (4.9)	8 (80)	7 (35)	7 (77.8)
GA (wk), mean ± SD	39.12 ± 1.3	34.07 ± 0.91	30.80 ± 1.1	26.30 ± 1.6	28.10 ± 2.2	27.33 ± 2.4
ROP, stages 1/2/3, *n*	0/0/0	0/0/0	0/0/0	0/0/0	13/19/4	0/2/14
Preeclampsia, *n* (%)	2 (3.4)	11 (20.4)	3 (7.3)	2 (20)	5 (25)	4 (44.4)
Placental insufficiency, *n* (%)	0 (0)	3 (5.6)	0 (0)	1 (10)	1 (5)	0 (0)
Maternal smoking, *n* (%)	2 (3.4)	1 (1.9)	4 (9.8)	0 (0)	2 (10.0)	2 (22.2)
HELLP syndrome, *n* (%)	0 (0)	4 (7.4)	1 (2.4)	0 (0)	2 (10.0)	0 (0)
Gestational diabetes, *n* (%)	0 (0)	3 (5.6)	1 (2.4)	1 (10)	1 (5.0)	0 (0)
Breastfeeding, *n* (%)	36 (61)	28 (51.9)	21 (51.2)	6 (60)	8 (40)	3 (33.3)

Among the male participants, 59 individuals had a GA of 37 weeks or more (group 1), 54 individuals had a GA between 33 and 36 weeks without ROP (group 2), 41 individuals had a GA between 29 and 32 weeks without ROP (group 3), 10 individuals had a GA of 28 weeks or less without ROP (group 4), 20 individuals had a GA between 24 and 32 weeks with ROP but without treatment (group 5), and nine individuals had a GA between 24 and 32 weeks with postnatal treatment for ROP (group 6) (Table 1). The recruitment efficacy for each group is displayed in Supplementary Figure S1.

### Descriptive Ocular Geometric Parameters of Female and Male Participants

A reduced anterior chamber depth and an increased lens thickness were noted in the ROP-treated group for both females and males. However, men showed longer or larger biometric features, including a deeper anterior chamber depth (*P* = 0.01), a bigger posterior segment (*P* = 0.02), a thicker central corneal thickness (*P* = 0.009), and a thicker central foveal thickness (*P* = 0.02) (Table 2).

**Table 2.** Ocular Geometric Parameters of the GPES Sample (*n* = 438) for Each Study Group Presented for Right Eyes and Female and Male Participants Separately

	Female Participants							Male Participants							
	Group							Group							
Parameter	1 (GA ≥ 37)	2 (GA 33–36)	3 (GA 29–32)	4 (GA ≤ 28)	5 (GA ≤ 32) ROP Without Treatment	6 (GA ≤ 32) ROP With Treatment	*P*	1 (GA ≥ 37)	2 GA (33–36)	3 GA (29–32)	4 (GA ≤ 28)	5 (GA ≤ 32) ROP Without Treatment	6 (GA ≤ 32) ROP With Treatment	*P*	Comparison by Sex, *P*
Participants/eyes, *n*	81/162	80/159	47/94	10/18	23/44	4/7		59/118	54/108	41/81	10/18	20/36	9/16		
Central corneal thickness (µm), mean ± SD	545. 69 ± 38.13	538.41 ± 30.45	537.19 ± 39.02	560.11 ± 41.93	550.50 ± 42.67	554.50 ± 25.21	0.29	557.12 ± 33.29	544.24 ± 31.03	549.20 ± 40.47	547.22 ± 46.52	555.00 ± 44.72	567.56 ± 48.60	0.31	0.009
Corneal radius (mm), mean ± SD	7.89 ± 0.33	7.71 ± 0.27	7.69 ± 0.33	7.71 ± 0.21	7.55 ± 0.31	7.83 ± 0.15	<0.001	7.89 ± 0.26	7.87 ± 0.27	7.73 ± 0.28	7.76 ± 0.19	7.73 ± 0.31	7.58 ± 0.27	0.003	0.02
Anterior chamber depth (mm), mean ± SD	2.88 ± 0.34	2.96 ± 0.31	3.01 ± 0.32	2.88 ± 0.35	2.93 ± 0.53	2.59 ± 0.31	0.21	3.01 ± 0.30	3.08 ± 0.32	3.08 ± 0.27	2.88 ± 0.28	3.08 ± 0.27	2.28 ± 0.78	<0.001	0.01
Lens thickness (mm), mean ± SD	3.78 ± 0.32	3.78 ± 0.34	3.78 ± 0.27	3.85 ±0.21	3.58 ± 0.22	4.16 ± 0.44	0.03	3.77 ± 0.35	3.79 ± 0.32	3.72 ± 0.27	3.66 ± 0.25	3.65 ± 0.30	4.61 ± 0.42	<0.001	0.90
Posterior segment length (mm), mean ± SD	16.97 ± 1.16	16.64 ± 1.11	16.65 ± 0.94	16.15 ± 0.72	16.23 ± 0.71	16.40 ± 1.10	0.02	17.14 ± 1.08	17.02 ± 0.97	16.48 ± 1.20	16.66 ± 0.88	17.23 ± 1.25	16.42 ± 2.50	0.05	0.02
Foveal retinal thickness (µm), mean ± SD	223.26 ± 16.14	237.16 ± 21.02	247.98 ± 30.16	263.75 ± 59.45	251.81 ± 40.07	276.00 ± 27.73	<0.001	223.78 ± 17.79	240.70 ± 19.17	252.64 ± 23.83	274.88 ± 30.04	275.07 ± 29.79	314.00 ± 42.75	<0.001	0.02

### Univariable and Multivariable Analyses

The univariable analysis showed a positive association between GA and BW percentile with mean corneal radius, whereas GA was negatively associated with central foveal thickness in female participants. The anterior chamber depth and lens thickness were not associated with GA or BW percentile (Table 3). Additionally, GA was positively associated with the posterior segment, whereas BW percentile was associated with central corneal thickness. Similar significant associations were observed in male participants, except that associations between GA and the posterior segment and between BW percentile and central corneal thickness were not evident.

**Table 3.** Linear Associations of Ocular Biometric Parameters With GA and BW Percentile (*n* = 438)

	Female Participants				Male Participants			
	Univariable Model		Multivariable Model		Univariable Model		Multivariable Model	
Parameter	*B* (95% CI)	*P*	*B* (95% CI)	*P*	*B* (95% CI)	*P*	*B* (95% CI)	*P*
Central corneal thickness (µm)								
GA (wk)	−0.021 (−1.1 to 1.05)	0.97	−0.033 (−1.090 to 1.024)	0.95	0.236 (−0.932 to 1.4)	0.69	0.042 (−1.171 to 1.255)	0.95
BW percentile	0.181 (0.006 to 0.356)	0.04	0.181 (0.007 to 0.356)	0.04	0.137 (−0.057 to 0.331)	0.17	0.135 (−0.065 to 0.336)	0.19
Mean corneal radius (mm)								
GA (wk)	0.019 (0.011 to 0.028)	<0.001	0.019 (0.011 to 0.027)	<0.001	0.016 (0.008 to 0.024)	<0.001	0.019 (0.011 to 0.005)	<0.001
BW percentile	0.003 (0.002 to 0.005)	<0.001	0.003 (0.002 to 0.005)	<0.001	0.003 (0.002 to 0.005)	<0.001	0.003 (0.002 to 0.005)	<0.001
Anterior chamber depth (mm)								
GA (wk)	0.005 (−0.01 to 0.011)	0.92	0.001 (−0.010 to 0.011)	0.92	0.004 (−0.008 to 0.015)	0.52	0.003 (−0.009 to 0.014)	0.65
BW percentile	−0.000 (−0.002 to 0.002)	0.74	−0.000 (−0.002 to 0.002)	0.74	0.000 (−0.002 to 0.002)	0.74	0.001 (−0.001 to 0.003)	0.40
Lens thickness (mm)								
GA (wk)	0.003 (−0.005 to 0.012)	0.43	0.003 (−0.005 to 0.012)	0.43	0.001 (−0.010 to 0.013)	0.8	0.002 (−0.009 to 0.013)	0.72
BW percentile	0.000 (−0.002 to 0.002)	0.99	−0.000 (−0.002 to 0.002)	0.98	−0.000 (−0.002 to 0.001)	0.68	−0.000 (−0.002 to 0.001)	0.63
Posterior segment length (mm)								
GA (wk)	0.051 (0.021 to 0.084)	0.001	0.050 (0.021 to 0.080)	0.001	0.026 (−0.013 to 0.066)	0.19	0.020 (−0.018 to 0.058)	0.31
BW percentile	0.003 (−0.002 to 0.008)	0.29	0.002 (−0.003 to 0.007)	0.34	0.006 (0.0 to 0.012)	0.06	0.005 (−0.001 to 0.011)	0.09
Central foveal thickness (µm)								
GA (wk)	−2.986 (−3.86 to −2.11)	<0.001	−2.987 (−3.859 to −2.115)	<0.001	–4.267 (−5.1 to −3.431)	<0.001	−4.271 (−5.166 to −3.383)	<0.001
BW percentile	−0.067 (−0.201 to 0.068)	0.33	−0.067 (−0.183 to −0.049)	0.25	−0.143 (−0.295 to 0.009)	0.065	0.004 (−0.128 to 0.127)	0.95

This is a sex-based analysis from the GPES.

In the multivariable model, both female and male participants had a larger mean corneal radius with higher GA, as well as a higher BW percentile. The posterior segment was positively associated with GA only in female participants. Central foveal thickness was negatively associated with GA in female participants and male participants.

### Multivariable Analyses With Effect Modification for Sex

The central foveal thickness was thinner with a higher GA in the multivariable model (Table 4). The displayed figure shows the raw data of central foveal thickness by gestational age for males and females (Fig.). Our further effect modification analysis reveals that at lower gestational age, males show a thicker central foveal thickness compared to females (*P* = 0.04). The sensitivity analysis incorporating age in the multivariable model yielded consistent results regarding the association between the effect modification of sex and GA and central foveal thickness (*B* = 1.347; 95% confidence interval [CI], 0.114–2.580; *P* = 0.03). This consistency was also observed in a multivariable model that additionally considered the presence of ROP and ROP treatment (*B* = 1.194; 95% CI, 0.015–2.373; *P* = 0.05). When foveal hypoplasia as an adjustment variable was included in the multivariable model, there was still a significant association of the effect modification between GA × sex and central foveal thickness (*B* = 1.026; 95% CI, 0.006–2.046; *P* = 0.05).

**Table 4.** Linear Associations of Ocular Geometric Parameters With GA and BW Percentile (*n* = 438)

	Multivariable Model	
	*B* (95% CI)	*P*
Central corneal thickness (µm)		
GA (wk)	0.037 (−1.176 to 1.251)	0.95
GA × sex	−0.069 (−1.679 to 1.540)	0.93
BW percentile	0.136 (−0.065 to 0.336)	0.18
BW percentile × sex	0.046 (−0.220 to 0.311)	0.74
Mean corneal radius (mm)		
GA (wk)	0.012 (0.004 to 0.020)	0.002
GA × sex	0.007 (−0.004 to 0.018)	0.22
BW percentile	0.003 (0.001 to 0.004)	0.001
BW percentile × sex	0.000 (−0.001 to 0.003)	0.56
Anterior chamber depth (mm)		
GA (wk)	0.003 (−0.009 to 0.015)	0.64
GA × sex	−0.002 (−0.018 to 0.013)	0.77
BW percentile	0.001 (−0.001 to 0.003)	0.40
BW percentile × sex	−0.001 (−0.004 to 0.002)	0.41
Lens thickness (mm)		
GA (wk)	0.002 (−0.009 to 0.013)	0.73
GA × sex	0.001 (−0.013 to 0.016)	0.84
BW percentile	−0.000 (−0.002 to 0.001)	0.62
BW percentile × sex	0.004 (−0.002 to 0.003)	0.74
Posterior segment length (mm)		
GA (wk)	0.020 (−0.019 to 0.058)	0.31
GA × sex	0.031 (−0.018 to 0.079)	0.22
BW percentile	0.005 (−0.001 to 0.011)	0.09
BW percentile × sex	−0.003 (−0.010 to 0.005)	0.52
Central foveal thickness (µm)		
GA (wk)	−4.275 (−5.166 to −3.384)	<0.001
GA × sex	1.288 (0.041 to 2.535)	0.04
BW percentile	0.004 (−0.120 to 0.128)	0.95
BW percentile × sex	−0.072 (−0.241 to 0.098)	0.40

This is a sex-based analysis from the GPES with incorporation of effect modification for sex. Adjustments were made for the effect of sex in each analysis (males were used as the reference group).

**Figure.**
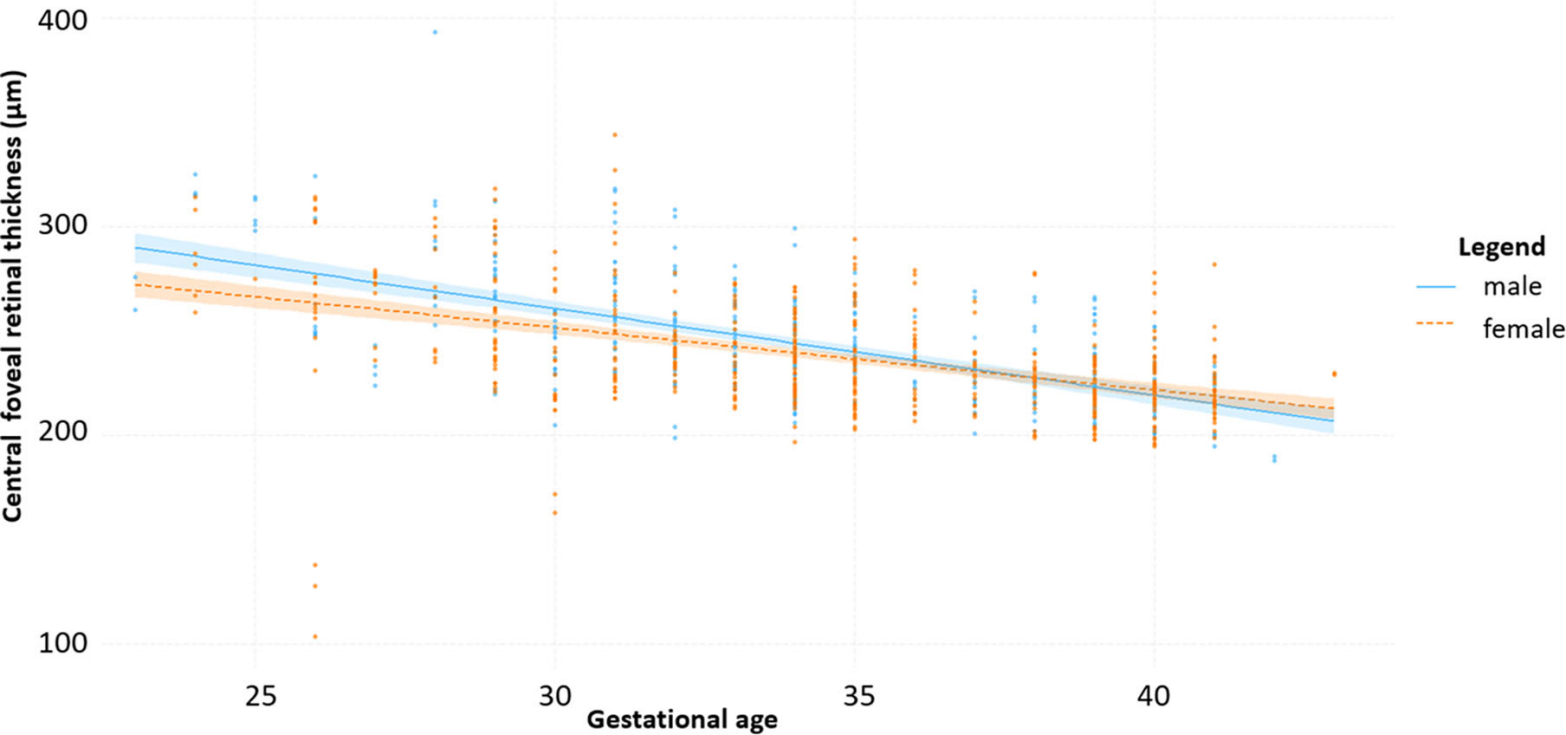
Relationship between gestational age and central foveal thickness by sex.

## Discussion

The present analysis provides new data on the long-term effects of GA and BW on ocular geometry in adults, with a primary emphasis on existing sex-specific differences. To our knowledge, this is the first study examining sex-specific differences in ocular biometry in individuals born preterm. The findings from our descriptive analysis highlight several notable differences in biometric measurements between male and female participants, with males generally having larger measurements, including a deeper anterior chamber depth, larger posterior segment, and thicker central foveal thickness. A prior investigation of the GPES showed that corneal morphology is influenced by GA and BW percentile.^19^ In addition to these findings, we now demonstrate how the associations between male and female participants differ.

The multivariable regression analyses of both female and male participants revealed a larger mean corneal radius with higher GA and BW percentile. The posterior segment was positively associated with GA, but this was only observed in female participants. Furthermore, the central corneal thickness was linked to the BW percentile exclusively in female participants. Central foveal thickness showed a negative association with GA in female and male participants.

The multivariable regression analyses with effect modification for sex on GA revealed a significant association with central foveal thickness (*B* = 1.288; *P* = 0.04) that was further supported by additional sensitivity analyses. Sex-specific differences related to prematurity were identified in other medical specialties, with higher mortality and morbidity among male preterm infants.^28^ The reasons for this may include the higher risk of metabolic and neurological complications in males.^29^ Furthermore, male preterm infants have a higher risk of developing ROP, intraventricular hemorrhage, and respiratory distress syndrome.^10^^,^^12^^,^^30^ These fundamental sex differences highlight the relevance of exploring additional aspects related to prematurity when sex variations may exist for a comprehensive evaluation of potential long-term consequences.

As there are no studies examining sex-specific differences in the eye biometry of preterm infants, we could not directly compare our results with matching studies; however, studies in adults showed comparable results related to generally larger biometric parameters in men.^4^^,^^5^ These biometric differences can be partly attributed to the distinct stature of women and men. When adjusting for these parameters (e.g., body height), sex-specific differences were no longer present.^31^ In contrast, one study adjusted for body height and the existing biometric sex differences remained, including longer axial length and larger vitreous chamber depth, as well as deeper anterior chamber depth in men.^4^

The association between corneal curvature and GA or BW percentile was present in both sexes. For posterior segment length, however, a relationship was solely observed for GA in female participants. An association between central corneal thickness and BW percentile was only observed in female participants, possibly due to the lower number of male participants.

In our descriptive analysis, we observed that, irrespective of gestational age, men generally show a thicker central foveal thickness compared to women. Wagner-Schuman et al. also showed a thicker central foveal thickness in men compared to women.^32^ This sex differences could be due to females possibly possessing a stronger centrifugal force at the macula, which could account for this phenomenon.^33^ Another observation was the relationship between GA and central foveal thickness, with a significant effect modification for sex, demonstrating that males have a thicker central foveal thickness at lower GA compared to females. Other studies have also shown a relationship between central foveal thickness and GA, with thinner central foveal thickness with higher GA.^26^^,^^34^^,^^35^ The reason for this could be that there is a disturbed inner retinal layer migration to the periphery after preterm delivery in preterm infants, resulting in a thicker central foveal thickness in preterm infants.^36^^,^^37^ Our results suggest that the effects of GA on central foveal thickness differ between women and men. A potential association could indicate fundamental differences in the development of central foveal thickness between the sexes in individuals born preterm. Furthermore, male individuals have an increased risk for severe retinopathy of prematurity,^38^^,^^39^ which is in line with our findings that retinal foveal morphology may be more vulnerable to prematurity in males. However, some studies do not indicate a connection between retinal thickness and an influence on visual acuity,^37^^,^^40^ in contrast with other studies that do,^26^^,^^34^ and this sex-specific difference indicates that the risk for lower visual acuity may be higher in males.

### Strengths and Limitations

This study is subject to several limitations. First, it is important to note that this was a single-center, hospital-based cohort study. We also encountered challenges in contacting several former newborns, and some participants opted not to take part in the study. Another limitation to consider is the relatively small number of participants with ROP and treated for ROP, which should be taken into account when interpreting our findings. Additionally, a limitation relates to the absence of lateral scaling in the OCT images, which could affect the accuracy of ocular geometry measurements. Despite these limitations, our study is the most extensive examination of adults born preterm at varying GAs. A comprehensive assessment of perinatal medical histories was meticulously conducted through the review of medical records, enabling us to analyze potential perinatal factors that may have influenced ocular geometry development. Furthermore, all measurements adhered to stringent standardized operating procedures to mitigate interrater variability stemming from individual examiners. To maintain objectivity, our investigators remained blinded to the participants’ birth-related characteristics throughout the study.

## Conclusions

In conclusion, our results demonstrate that biometric parameters differ between the sexes, with larger biometric parameters in male participants. The known associations with GA, however, are not influenced by sex, except for a hint of an association with central foveal thickness, indicating disparities in the developmental trajectory of the macula in relation to GA. A thicker central foveal thickness could affect the visual acuity of individuals born preterm in adulthood, with a more pronounced influence in male individuals.

## Supplementary Material

Supplement 1
