# Supplementary figures and images for: Sex-Specific Differences in the Relationship Between Prematurity and Ocular Geometry
Source: Invest Ophthalmol Vis Sci. 2024 Jun 14;65(6):23. doi: 10.1167/iovs.65.6.23 (PMC11182371; doi:10.1167/iovs.65.6.23)

# Study Design

## Gutenberg Prematurity Study (GPS)

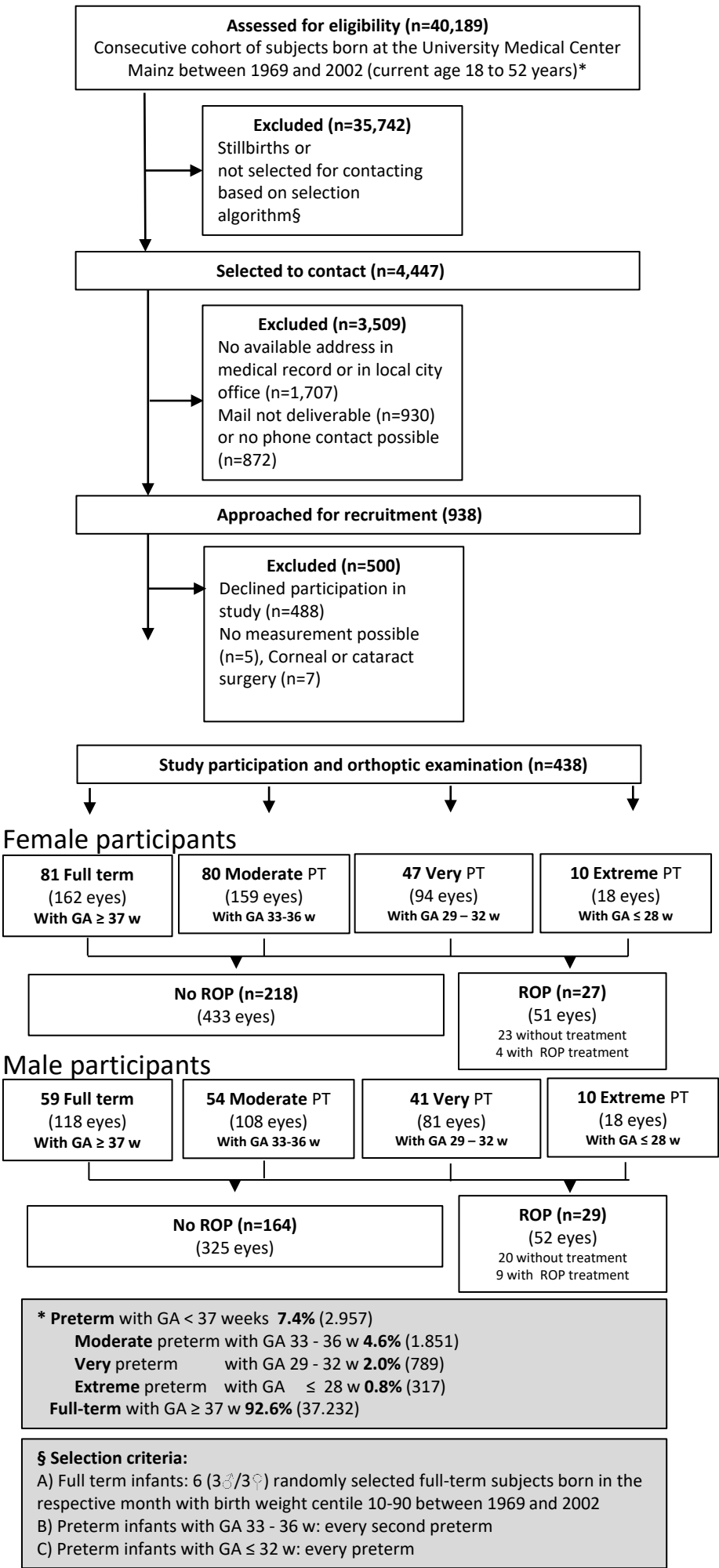

Supplement: Supplement 1 [file iovs-65-6-23_s001.pdf]
